# Supplementary material for: Maximal rectification ratios for idealized bi-segment thermal rectifiers
Source: Sci Rep. 2015 Aug 4;5:12677. doi: 10.1038/srep12677 (PMC4523837; doi:10.1038/srep12677)
Supplement: Supplementary Information [file srep12677-s1.pdf]

Maximal rectification ratios for idealized bi-segment thermal rectifiers

Tien-Mo Shih[1,2,a)] Zhaojing Gao[1], Ziquan Guo[3], Holger Merlitz[1,4], Patrick J. Pagni[5], and Zhong Chen[3b)]

(Dated: 13 April 2015)

[a)]Correspondence to [tmshih@xmu.edu.cn]

[b)]Correspondence to [chenz@xmu.edu.cn]

SUPPLEMENTARY INFORMATION

S-1 Derivation of $\psi_i = 0.5(\psi_{i-1} + \psi_{i+1})$

Consider a grid, on which regular node $i-1$, halfway node w (denoting "west to node i"), and regular node i lie. At the halfway node w, the heat flux in the segment A can be written as

$$J_w = -\kappa_w \left(\frac{dT}{dx}\right)_w = (d_1 + 2d_2T_w)\frac{T_{i-1} - T_i}{\Delta x}. \qquad (S-1)$$

If Δx is taken to be infinitesimal, then $T_w \approx (T_{i-1} + T_i)/2$. Hence,

$$\begin{aligned} J_w &= [d_1 + d_2(T_{i-1} + T_i)](T_{i-1} - T_i)/\Delta x \\ &= d_1(T_{i-1} - T_i) + d_2(T_{i-1}^2 - T_i^2) \qquad (S-2) \end{aligned}$$

or

$$J_w = \psi_{i-1} - \psi_i, \qquad (S-3)$$

where $\psi = d_1T + d_2T^2$. Likewise $J_e = \psi_i - \psi_{i+1}$. Since $J_w = J_e$ must hold in the steady state, we obtain $\psi_{i-1} - \psi_i = \psi_i - \psi_{i+1}$, or $\psi_i = 0.5(\psi_{i-1} + \psi_{i+1})$.

S-2 Derivation of Eq.(6)

$$R = \frac{\psi_{HA} - \psi_{pA}}{-\psi_{LA} + \psi_{qA}} = \frac{d_1T_H + d_2T_H^2 - d_1p - d_2p^2}{-d_1 - d_2 + d_1q + d_2q^2}$$

$$= \frac{d_1(T_H - p) + d_2(T_H^2 - p^2)}{d_1(q - T_L) + d_2(q_2 - T_L)} = \frac{(d_1 + d_2T_H + d_2p)(T_H - p)}{(d_1 + d_2 + d_2q)(q - T_L)}.$$

Defining $\kappa_1 = d_1 + d_2(T_H + p)$ and $\kappa_2 = d_1 + d_2(T_L + q)$, we can obtain

$$R = \frac{\kappa_1(T_H - p)}{\kappa_2(q - T_L)}.$$

S-3 Explanation of the difference between the Newton-Raphson method and the modified version of the Newton-Raphson method

In the official Newton-Raphson method, all nonlinear terms are faithfully linearized using Taylors series expansion. In the modified version, for the purpose of avoiding extremely tedious algebraic manipulations, some nonlinear terms are temporarily treated as constants

and not linearized. During iterations combined with under-relaxation, these terms are moved to the right-hand side of equations. If the solution fortunately converges, much tedious algebraic work is successfully avoided. If otherwise, perhaps the official Newton-Raphson method must be reluctantly used. In the present case, all solutions aided with the under-relaxation did converge fortunately.
